# Medical cannabis authorization and opioid milligram equivalents over time in patients with chronic pain: a retrospective analysis

**DOI:** 10.1093/pm/pnaf113

**Published:** 2025-08-21

**Authors:** Michelle Sexton, Nicholas C Glodosky, Michael Cleveland, Carrie Cuttler, Euyhyun Lee, Gregory R Polston, Timothy Furnish, Imanuel Lerman, Nathaniel M Schuster, Mark S Wallace

**Affiliations:** Centers for Integrative Health, University of California San Diego, San Diego, CA 92037, United States; Department of Psychology, Washington State University, Pullman, WA 99164, United States; Department of Psychology, Washington State University, Pullman, WA 99164, United States; Department of Psychology, Washington State University, Pullman, WA 99164, United States; Altman Clinical and Translational Research Institute, University of California San Diego, San Diego, CA 92093, United States; Department of Anesthesiology, Division of Pain Medicine, University of California San Diego, San Diego, CA 92037, United States; Department of Anesthesiology, Division of Pain Medicine, University of California San Diego, San Diego, CA 92037, United States; Department of Anesthesiology, Division of Pain Medicine, University of California San Diego, San Diego, CA 92037, United States; Department of Anesthesiology, Division of Pain Medicine, University of California San Diego, San Diego, CA 92037, United States; Department of Anesthesiology, Division of Pain Medicine, University of California San Diego, San Diego, CA 92037, United States

**Keywords:** cannabis, chronic pain, opioids, morphine milligram equivalents, opioid-sparing, opioid-tapering

## Abstract

**Objective:**

Strategies are needed for patients with chronic pain who are using opioids to safely and effectively wean opioids without worsening of pain. The objective was to measure associations between medical cannabis authorization (MCA) and opioid milligram equivalents (OME) in patients with chronic non-cancer pain.

**Design:**

A longitudinal, retrospective cohort analysis from July 2016 to August 2019.

**Setting:**

Electronic health record data were analyzed.

**Subjects:**

Adult patients (≥18 years) seen in a university-based pain clinic.

**Methods:**

Longitudinal multilevel modeling with maximum likelihood estimation.

**Results:**

Average overall OME at the final time point was 33.4 mg/day (*SE *= 1.18) with increase over time of 0.45 mg/day per quarter (not statistically significant). Average OME in those without MCA was 32.60 mg/day (*SE *= 1.11) versus 38.51 mg/day (*SE *= 4.81) in those with MCA, not significantly different. Medical cannabis consultation predicted a nonsignificant decrease of 14.25 mg/day OME. Long-term opioid use was a significant predictor with a mean OME of 85.34 mg/day, 63 mg/day higher than the rest of the cohort at the final quarter (*t *= 5.77, *SE *= 10.93, *P* < 0.0001).

**Conclusions:**

In this longitudinal study of electronic health record data, MCA was not associated with a statistically significant decrease in OME over time. However, patients with long-term opioid use diagnostic code demonstrated a significantly higher endpoint OME. Future prospective research is needed to establish whether there are opioid-sparing effects of cannabis in humans.

## Teaser

Strategies for patients with chronic pain to safely and effectively wean opioids, without worsening of pain, may include a trial of cannabis. The study objective was to measure associations between medical cannabis authorization and opioid milligram equivalents in patients with chronic non-cancer pain. We did not find that cannabis authorization by the pain doctor was associated with a reduction in OME over time. Cannabis dose consultation and tapering schedules could improve this outcome.

Tweet: Does cannabis actually help when exiting opioid therapy for chronic pain? Mixed results in the literature, and in this study from a major academic center, show the evidence is weak. Cannabis dosing and administration consultations with monitored opioid tapering could change this outcome.

## Introduction

While initiating opioids for chronic non-cancer pain has become less common over the past decade, millions of Americans with chronic non-cancer pain have been on opioid therapy for decades.^1^ Long-term opioid use (LTOU) may or may not improve chronic pain or function, may co-occur with other substance use disorders, and is associated with elevated anxiety and depression symptoms.^2–5^ In 2019, 22.1% of adults in the United States with chronic pain were prescribed opioids in the previous 3 months.^6^ Similarly, 30% of older adults self-reported use of prescription opioids in Brazil during the previous 3 months.^7^ These high prevalence rates are concerning as prescription opioid use can lead to hyperalgesia, tolerance, opioid use disorder, and increased risk of death.^1^^,^^8^ The use of cannabis for medical purposes was approved by voter referendum in California in 1996. The California State Department of Health provides an official medical cannabis authorization (MCA) form for physicians to issue to patients, signed by the doctor and stored in the electronic health record (EHR) at the University of California San Diego (UCSD).

Since the 1990s, there have been well-described roles for the endocannabinoid system as a mechanistic target for treating pain.^9–11^ The opioid and endocannabinoid systems share neuroanatomical, neurochemical, and pharmacological characteristics and co-stimulation of these systems reputedly has opioid-sparing effects.^12^^,^^13^ Specifically, delta-9 tetrahydrocannabinol (THC), a high affinity cannabinoid 1 receptor partial agonist, may enhance opioid receptor signaling.^11^^,^^14^ Consistent with this, a 2017 meta-analysis of preclinical studies revealed that THC administered with morphine had opioid-sparing effects.^13^ However, low-certainty to mixed evidence has been found for opioid-sparing effects in human studies.^13^^,^^15^ Recent data from the New York State Prescription Monitoring Program measured opioid milligram equivalents (OME) in patients who received medical cannabis, suggesting that medical cannabis use in patients receiving higher opioid dosages at baseline, and for longer duration (>30 days), was associated with reduction in OME.^16^

Surveys and naturalistic examinations have revealed self-reported pain reductions in the short term by cannabis users.^17^^,^^18^ These users have also reported cannabis serving as a substitute for medications such as opioids.^19–21^ Notably, there is some evidence for cannabis efficacy for neuropathic pain which represents a broad category of diagnoses with an unmet clinical need.^22^ A survey analysis of 244 Michigan cannabis dispensary visitors with MCA, who endorsed centralized pain, a type of neuropathic pain, measured a 64% reduction in self-reported opioid use.^23^ A 2021 meta-analysis of randomized controlled trials and observational data found a low certainty of evidence that medical cannabis had any effect on prescribed opioid use.^24^ Another systematic review and meta-analysis found few good quality randomized controlled trials that have explored cannabis substitution effects, indicating a pressing need for additional high-quality trials.^25^

While some states have medical cannabis registry systems, healthcare systems may not have routinely integrated MCA or medical cannabis consultations into their health records.^26^ An authorization is a written documentation of a healthcare practitioner’s finding that medical use of cannabis is appropriate for the patient, while a consultation is an education-based consultation on administration forms and dosing. Based on a pilot case series of patients who received MCA and a cannabis consultation and successfully tapered opioids, we hypothesized that the MCA would be associated with decreased OME over time (Supplement 1: Appendix S1). In this study, we assessed associations between MCA issued by the pain physician and calculated OME over time.

## Study design and setting

A longitudinal design was used to analyze archival EHR data collected between July 2016 and August of 2019. This study followed the Strengthening the Reporting of Observations Studies in Epidemiology reporting guidelines. In accordance with the common rule, the study received a waiver of informed consent from the UCSD Office of IRB Administration. All identifying information was removed from records to conceal patient identity. A Certificate of Confidentiality was obtained from the National Institute of Drug Abuse due to the nature of sensitive information collected during the course of the study.

The archival data were obtained from a large, university-based pain medicine center in San Diego, California that serves around 13 000 patients annually in ambulatory and hospital settings. During the period that records were generated, federal and local policies were undergoing rapid changes due to a 5-fold increase in opioid-related deaths in 2016 compared to 1999.^27^ The pain specialists were following Centers for Disease Control and Prevention guidelines for prescribing opioids for chronic pain. Patients may have been referred during this time period to the pain center from previous opioid prescribers. The Medical Board of California issued guidelines for recommendation of medical cannabis in 2018.^28^ The physicians had agreed-upon guidelines for issuance of MCA to chronic pain patients (Supplement 1: Appendix S2). The study is a retrospective chart review, valuable for directing subsequent prospective investigations.^29^

### Data source

The Data Extraction Concierge Service at the Altman Center for Translational Research Institute at UCSD generated the patient list. This service allows for extraction of pre-defined, patient-level datasets from the EHR for clinical research purposes, which reduces transcription error and limits any interpretation by coders (Supplement 1: Appendix S3).

**Figure 1.**
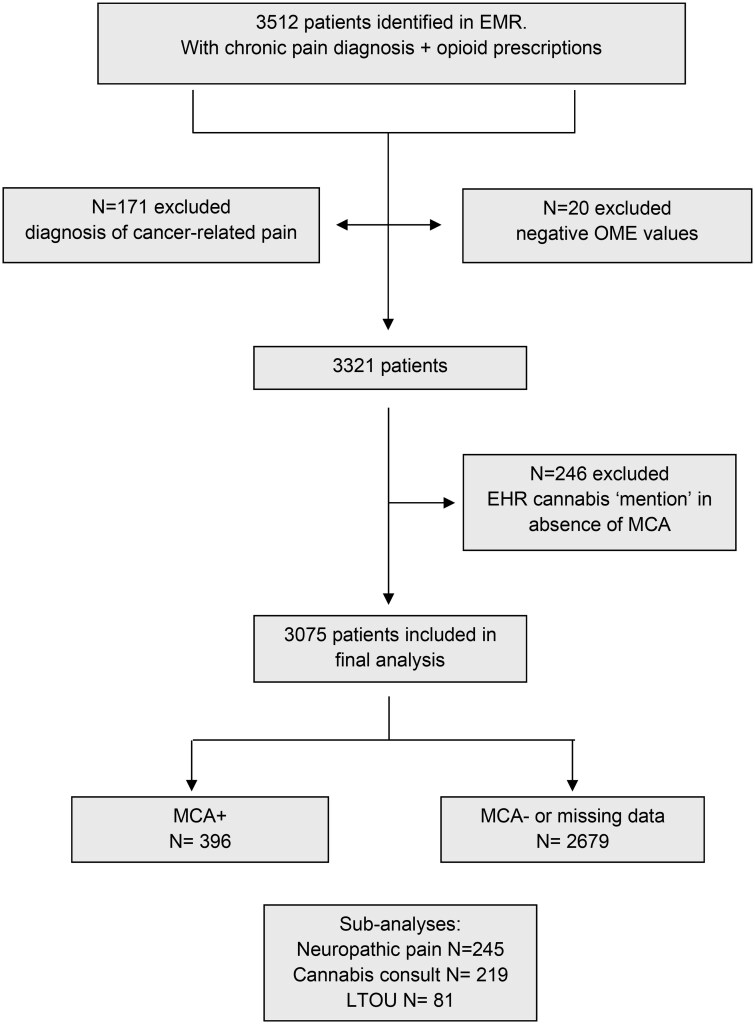
Flow chart for study population 2016-2019.

The cohort was defined as having record of appointments at the pain clinic during the time period, and opioid prescriptions in the UCSD system. Tramadol was not included as an opioid prescription due to findings that it has low abuse potential.^30^ Data on record of MCA and presence/absence of a cannabis consultation were retrieved. Records were accessed for 3512 adults (≥18 years of age) and 3075 patients were included in the final analysis (see Figure 1).

#### Exposure definition

Presence of diagnostic codes for chronic or neuropathic pain (Supplement 2: Appendix S1), issuance of diagnostic code for long-term (current) opioid use (LTOU; Z79.891), MCA, and presence or absence of a cannabis consultation (Supplement 1: Appendix S4) were the primary predictor variables of interest. The cannabis consultation was provided by a licensed doctor and delivered education on administration options and therapeutic dosing.^31^^,^^32^

#### Exclusion criteria

Exclusion criteria included: <18 years of age; diagnostic code related to cancer-related pain or paraneoplastic neuropathy (due to opioid weaning being discouraged in these populations); calculated OME values that were negative for reasons described below (Supplement 2: Appendix S2); and patients with mention of non-medical cannabis use in the patient chart (due to lack of clarity on intended use and the potential for comorbid cannabis use disorder).

#### Main outcome definition

The primary outcome measure, planned prior to data collection, was change in OME over time. The a priori hypothesis was that MCA for patients with prescribed opioids would be associated with reductions in OME over time.

### Methods

#### OME calculation

Opioid prescriptions were converted to OME, based on CDC conversion guide per day, per quarter.^33^ Negative OME values were calculated when the prescription “end date” was before the “start date” (system errors) and were excluded from the analysis (Supplement 2: Appendix S2).

#### Statistical analyses

Longitudinal multilevel modeling (MLM) with Maximum Likelihood Estimation was used to analyze the data (SAS version 9.4 PROC MIXED: SAS Institute Inc., 2013).^34^ MLM allowed for examining changes in OME within patients, while also allowing for missing waves of data without excluding patients for partial data. MLM was specifically chosen as an analytic approach because it is able to account for MCA issuance at different time points and estimate change over time.^35^ If no record of an opioid prescription existed for a patient in any quarter, OME was assumed to be zero.

A baseline unconditional growth model was created containing only the outcome and a single predictor of linear time. This model described the average calculated OME and rate of change for all patients at the final wave of data collection and served as a comparison for conditional models. We then estimated a conditional model with predictors of OME including the average effect of MCA as well as the effects of cannabis consultation and presence of diagnostic code for LTOU. We also examined the effects of these predictors using a subgroup of patients with diagnoses specific for neuropathic pain. In all models, time was centered at the final wave of data collection (Quarter 13) so that the intercept represents the most recent timepoint. This timepoint was chosen to represent results at “end of the treatment observation period”; however, it should be noted that this timepoint may not represent end of treatment for all patients and may represent the initiation of treatment for some.

## Results

### Demographics and patient characteristics

Among the 3075 patients included in the final analysis, age ranged from 24 to 90 years of age with an average age of 60.7 (*SE *= 0.26); 83.74% were White and 61.63% were female. 9.85% of patients had a diagnostic code for neuropathic pain; 12.88% of patients were issued MCA; and 2.63% had a LTOU diagnostic code. Overall, the cannabis consultation was accessed by 57% of those issued MCA (Table 1). 5.3% of patients in the study with an LTOU diagnostic code received MCA and accessed the cannabis consultation, while 14% of patients with neuropathic pain diagnoses completed the consultation.

**Table 1.** Overall sample characteristics.

	Overall sample (*n *= 3075)		MCA− (*n *= 2216)		MCA+ (*n *= 396)	
Characteristic	*M* (*SE*)	Range	*M* (*SE*)	Range	*M* (*SE*)	Range
Age	60.71 (0.26)	22-97	60.43 (0.28)	22-97	62.54 (0.73)	24-90
OME	33.47 (1.18)	0-11 640	32.60 (1.11)	0-7907.39	38.51 (4.81)	0-11 640
	%		%		%	
White	83.74%		83.34%		86.40%	
Female	61.63%		61.66%		63.73%	
Neuropathic pain DX	9.85%		8.57%		13.89%	
LTOU	2.63%		2.57%		5.30%	
MCA	12.88%					

*n = 463* patients had missing values for MCA. MCA−, patients with no EHR record of receiving medical cannabis authorization; MCA+, patients with EHR record of receiving medical cannabis authorization.

### OME change over time

Table 2 presents the results of the baseline and conditional MLM models, using time as the only predictor. The baseline model showed that the average calculated OME prescription at the final timepoint was 26.91 mg/day. There was a nonsignificant increase in the OME over time of 0.45 OME per quarter (t = 1.62, SE = 0.28, *P* = 0.106).

**Table 2.** Results of multilevel models with OME as outcome with time as a predictor.

	Baseline model			Final model		
Effect	Estimate	*SE*	*P*	Estimate	*SE*	*P*
Intercept	26.91	2.72	< 0.0001	22.30	3.30	< 0.0001
Time	0.45	0.28	0.106	0.08	0.34	0.817
MCA				+12.75	8.29	0.124
MCA by time				+1.37	1.48	0.354
Dosing consultation				+6.37	7.90	0.420
LTOU				+63.04	10.93	< 0.0001
−2 Log likelihood	238 431.4			208 370.0		
AIC	238 443.4			208 390.0		
BIC	238 479.6			208 448.7		

Baseline model represents an unconditional growth model containing only time as a predictor and the final model includes the effects of MCA, dosing consultation, and LTOU on the intercept and MCA over time. AIC, Akaike Information Criterion; BIC, Bayesian Information Criterion.

**Figure 2.**
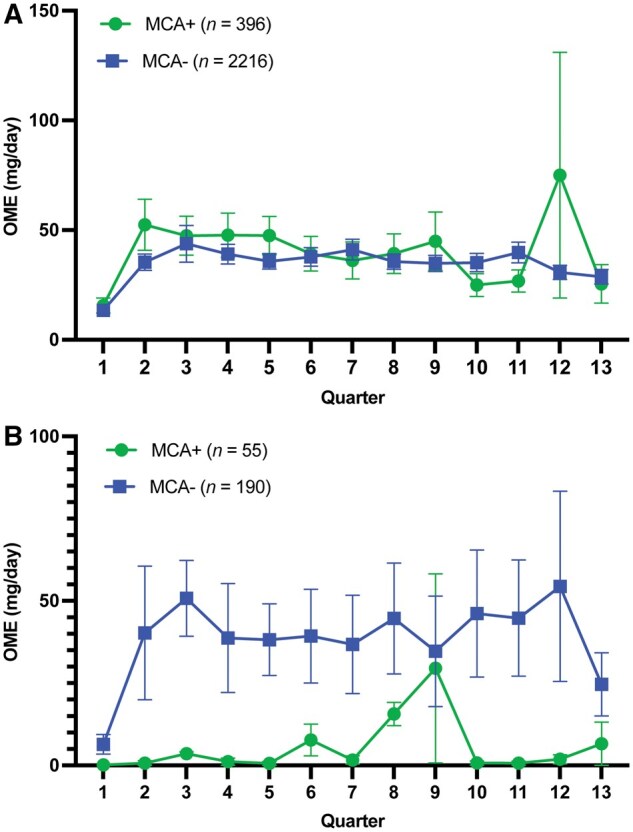
(A) Change in OME for patients with and without MCA. Results from the MLM indicated that patients without MCA (*n *= 2216) had lower OME (not statistically significant: *P* = 0.124) compared to those with MCA (*n *= 396). Similarly, MCA was associated with a greater increase of OME over time, but this was also not statistically significant (*P* = 0.354). *MCA, medical cannabis authorization; OME, opioid milligram equivalents. (B) Change in OME for patients with neuropathic pain. Results indicate that among patients with neuropathic pain, those without MCA (*n *= 248) had higher OME (not statistically significant: *P* = 0.818) compared to those with MCA (*n *= 55). Similarly, MCA was associated with a lower increase of OME over time, but this was also not statistically significant (*P* = 0.785). *MCA, medical cannabis authorization; OME, opioid milligram equivalents.

In the final model, patients with MCA had an average OME of 35.05 mg/day, while patients without MCA had a lower average OME of 22.30 mg/day (excluding those with LTOU). Changes in OME over time did not significantly differ between those with and without MCA (estimate = 1.37, t = 0.93, SE = 1.48, *P* = 0.354) (Figure 2A). Those with MCA had baseline OME of 17.65 mg/day while those without MCA had baseline OME of 21.34. At the final timepoint, those with MCA had OME of 35.05 mg/day compared to 22.30 mg/day for those without MCA. However, patients with the LTOU diagnostic code had a significantly higher average OME of 85.34 mg/day, compared to 22.30 mg/day in patients without the LTOU code (t = 5.77, SE = 10.93, *P* < 0.0001).

In a secondary exploratory analysis (Table 3), we analyzed results for patients diagnosed specifically with neuropathic pain. Models were adjusted to include predictors for MCA, cannabis consultation, and LTOU diagnostic codes. The baseline unconditional growth model indicated that, on average, patients with neuropathic pain had an OME prescription of 20.75 mg/day at the final timepoint. There was no significant increase in OME over time (estimate = 0.96, t = 1.46, SE = 0.65, *P* = 0.145).

**Table 3.** Results of multilevel models with OME as outcome for patients with neuropathic pain.

	Baseline model			Final model		
Effect	Estimate	*SE*	*P*	Estimate	*SE*	*P*
Intercept	20.75	6.80	0.003	23.82	9.28	0.011
Time	0.96	0.65	0.145	1.10	0.87	0.207
MCA				−4.53	19.72	0.818
MCA by time				−0.97	3.58	0.785
Dosing consultation				−14.25	15.04	0.344
LTOU				81.13	34.79	0.021
−2 Log likelihood	14 028.0			11 881.7		
AIC	14 040.0			11 901.7		
BIC	14 062.3			11 936.8		

Baseline model represents an unconditional growth model containing only time as a predictor and the final model includes the effects of MCA, dosing consultation, and LTOU on the intercept and MCA over time. AIC, Akaike Information Criterion; BIC, Bayesian Information Criterion.

Furthermore, MCA had no significant effect on OME for those with neuropathic pain (estimate = -4.53, t = -0.23, SE = 19.72, *P* = 0.818), nor did it significantly impact the change in OME over time (estimate = -0.97, t = -0.27, SE = 3.58, *P* = 0.785). Those with MCA received 17.73 mg/day OME at baseline while those without MCA had OME of 10.62. At the final timepoint, those with MCA received OME of 19.29 mg/day compared to 23.82 mg/day for those without MCA (Figure 2B). Cannabis consultation predicted a nonsignificant decrease of 14.25 mg/day OME (t = -0.95, SE = 15.04, *P* = 0.344) for those with neuropathic pain. LTOU diagnostic code was associated with a significantly higher OME of 104.95 mg/day at the final timepoint, which was 81.13 mg/day higher than for those without the LTOU code (t = 2.33, SE = 34.79, *P* = 0.021).

## Conclusions and relevance

To our knowledge, this is the first study analyzing associations between MCA issuance and OME in a university-based pain clinic. These data provide another context to both the association found between medical cannabis laws and decrease in opioid overdose mortality reported from 1999 to 2010, and a subsequent analysis that reported a reversal of that trend.^36–38^ Results of analyses of archival patient-level data comparing changes in OME over time for patients with versus without MCA revealed no significant differences. Patients with MCA that accessed cannabis consultations had a nonsignificant decrease in OME over time, particularly for patients with a neuropathic pain diagnosis.

As a contrast, an increase in OME was found for patients with a diagnostic code for LTOU (Z79.891), irrespective of MCA. This Z code may be used to indicate LTOU or for billing purposes to monitor opioids with urine drug screening. While this code *may* suggest a more complicated chronic opioid user or misuse, we did not assess for codes used for uncomplicated opioid use (F11.9), opioid abuse (F11.1), or opioid dependence (F11.2). F11.9 has been found to be the most referenced method to document opioid misuse.^39^ We did not retrieve data on urine drug screening, structured screening tools or clinical observation that may have provided additional information on this patient population.

In this setting, quarterly OME calculations for persons on chronic opioid therapy and/or with LTOU diagnostic code did not conform to other studies that reported associations between medical cannabis use and reductions in OME.^16^^,^^40^ These results are in line with the findings of Martins et al, where States with recreational *and* medical cannabis laws showed few benefits and an inconsistent pattern of decreased odds of any opioid outcomes.^41^ Furthermore, a systematic review and meta-analysis by Nielsen et al concluded from 3 high-quality RCTs for chronic non-cancer pain using dronabinol and 2 using smoked cannabis that analgesic outcomes were conflicting, only one measuring opioid use, with no change. In contrast, the observational studies found that 39% of the included studies (n = 8) reported opioid cessation and 85% (n = 7 studies) reported reduction.^42^ Another recent systematic review concluded that attention to cannabinoid dosing is an area that needs attention, an important point that we attempted to address in this study by providing the dosing consultation.^43^ By increasing the percentage of patients receiving the consultation, combined with specific tapering instructions, this outcome could be improved.

Observational and prospective studies report that cannabis is commonly used for pain relief or for opioid substitution with mixed results.^17^^,^^44–49^ These studies, from survey level data to prospective controlled trials, illustrate that the heterogeneity in the type of cannabis used, the length of the intervention, size of the study, and methodology may confound meta-analysis. Evidence for opioid-sparing effects of cannabis use in humans is present in the literature; however, it is scant and inconsistent.^50–52^ The functional interactions between opioid and endocannabinoid systems are complex and still not fully understood, including changes in the functionality of both systems.^53^ While the cannabinoid 1 receptor has been shown to be a modulator of opioid reward (proposed to serve as an anti-reward), these results do not support that cannabis authorization, particularly for persons with a diagnostic code for LTOU, predicts opioid-sparing benefit.^54^^,^^55^ These results also support the findings of Hurley et al that consensus and validation on the use of opioid use diagnostic codes is needed.^39^

The case series pilot data along with previous literature drove the hypothesis for this research: MCA, coupled with a cannabis consultation, may be negatively associated with OME. Some evidence supports short-term, low-dose cannabis for safely and effectively reducing neuropathic pain and evidence that there may be a therapeutic window for THC to provide pain relief.^43^^,^^56^^,^^57^ Furthermore, people report they are using cannabis for chronic pain and may be substituting this for other medications, including opioids.^58^ Limitations of the EHR did not allow for assessing pain scores over time and other patient-reported outcomes (functional, mood, etc) were not consistently available.

Prospective controlled studies are required to confirm or negate whether cannabis, in any form or dose, can spare opioid use in patients living with chronic pain and using prescribed opioids. Specific for this data, vaporized cannabis was shown to have reinforcing properties in rodents, which could implicate that people at-risk for addiction may or may not benefit from receiving MCA and/or be at risk for developing a substance use disorder.^59^ At the same time, studies of inhaled cannabis for neuropathic pain show efficacy for, and may augment, pain relief.^45–48^^,^^56^^,^^60^^,^^61^ It is an important finding that there was a trend for decrease in OME in patients who accessed the cannabis consultation, while there was a low incidence of those with LTOU accessing the consultation.

Clinicians considering providing MCA for chronic pain and/or to replace or complement opioids should consider assessing risk of cannabis use disorder, and to carefully consider which diagnostic code for opioid use may apply when issuing MCA to patients with LTOU. Patient education, including cannabis consultation for education on dosing and administration, along with opioid tapering schedules, is warranted.^62^^,^^63^ Follow-up using compassionate inquiry into the amount and frequency of cannabis and opioid use, along with clinical guidance to assure adherence to opioid tapers, could help to minimize risk for substance use disorders and maximize tapering success.

Strengths of this study include that this is the largest of this type of investigation, a patient population sampled through an academic pain center, not at the dispensary level, an approach which may have reduced selection bias for cannabis use.^23^^,^^64–66^ The large sample size and patient-level data from the EHR provided precise information on diagnostic codes. The use of MLM for examining changes in OME within patients across time can account for the natural nesting of multiple observations within individuals, varying timepoints of MCA issuance while also allowing for unbalanced or missing waves of data without excluding patients for partial data.^35^

### Limitations

The findings of this study should be interpreted in the context of several limitations. While MCA was clearly documented (as provided by the pain physician: outside authorizations would not have been identified), it is unclear whether this resulted in any cannabis use; over what timeframe cannabis was used; the methods of cannabis administration; or the dose/frequency of cannabis use. We did not measure pain scores, allowing for the potential that patients may have received pain benefit from adding cannabis to their opioids despite not tapering. An assumption was made that the opioid prescription was filled as written; however, we were not permitted access to state-level prescription data for opioids for this cohort, leaving the implication that OME may have been different from what was calculated. There was also the potential that the EHR had duplicate records for a prescription or that the prescription duration was outside of the quarter range. All physicians may not have used the LTOU diagnostic code in the same way and we did not assess for use of other codes that could indicate use of opioids, opioid abuse, or opioid dependence. Finally, findings from this study are based on a sample of patients enrolled in a large health care system who were seen in the Pain Division and thus might not be generalizable to patients in other health systems, especially those where adult or medical cannabis use is not legal.

### Conclusions

This longitudinal study found no significant association between MCA and OME over time. However, there was a trend suggesting reduction in OME in subjects receiving MCA when coupled with a dosing consultation. Additionally, there was an association of LTOU diagnostic code with an increase in OME over time, irrespective of presence or absence of MCA. Based on these results, clinicians considering MCA for patients with chronic pain should focus on goals of providing patients with tapering schedules and monitoring pain/functional scores at each visit and consider the value of providing cannabis consultations for medical cannabis use. Coordination with opioid prescribers—when not the pain specialist—could facilitate opioid tapering. Ultimately, the risk/benefit profile of recommending medical cannabis for pain in patients on long-term opioid therapy likely requires careful consideration along with multidisciplinary approaches.^67^ While some evidence suggests that medical cannabis may decrease opioid use, these findings emphasize the need for further prospective trials to assess the impact of medical cannabis use on opioid use and pain management in patients with chronic pain.

## Supplementary Material

pnaf113_Supplementary_Data
