# Supplementary material for: Medical cannabis authorization and opioid milligram equivalents over time in patients with chronic pain: a retrospective analysis
Source: Pain Med. 2025 Aug 21;27(2):127–35. doi: 10.1093/pm/pnaf113 (PMC12865101; doi:10.1093/pm/pnaf113)
Supplement: pnaf113_Supplementary_Data [file pnaf113_supplementary_data.zip › Supplement 1.docx]

**Supplemental Online Content**

Medical Cannabis Authorization and Opioid Milligram Equivalents Over Time In Patients with Chronic Pain: A Retrospective Analysis

Michelle Sexton ND^1^, Nicholas Glodosky PhD^2^, Michael Cleveland PhD^2^ , Carrie Cuttler PhD^2^, Euyhyun Lee^3^, Greg Polston MD^4^, Tim Furnish MD^4^, Emanuel Lerman MD^4^, Mark Wallace MD^4^, Nathaniel Schuster MD^4^

eAppendix 1: Preliminary Results from Case Series

eAppendix 2: General Guidelines used for issuance of MCA to patients

eAppendix 3 Data Dictionary for Chart extraction

eAppendix 4 General Guidelines used for Dosing Consultation

This supplemental material has been provided by the authors to give readers additional information about their work.

**eAppendix 1**: **Hypothesis-driving pilot data**

| **Subject** | **AGE** | **Diagnosis** | **OME** | | **Subjective Pain score (average)** | |
| --- | --- | --- | --- | --- | --- | --- |
|  | | | **Before** | **After** | **Before** | **After** |
| Male | 72 | Failed back surgical syndrome | 220 | 40 (8-wk) | 9/10 | 4/10 |
| Female | 66 | Chronic LBP | 42.5 | 0 (4-wk) | 7/10 | 0/10 |
| Female | 66 | Failed back surgical syndrome | 30 | 0 (4-wk) | 7/10 | 3/10 |
| Male | 73 | Spinal stenosis/radiculopathy | 135 | 30 (8-wk) | 7/10 | 5/10 |
| Female | 50 | Cervical Spondylosis | 69 | 22.5 (4-wk) | 5/10 | 4/10 |
| Male | 61 | Failed Back surgical syndrome | 120 | 15 (4-wk) | 8/10 | 5/10 |

Average reduction in pain score = 3.6

Average decrease in OME = 85.58%

**Background:** Six patients with a diagnosis of chronic neuropathic pain, seen in the Department of Anesthesiology, Division of Pain Medicine at the University of California San Diego. All six were issued authorizations for medical cannabis authorizations (MCA) to facilitate pain relief and opioid tapering.

**Methods:** Data from the EHR provided the information for the case series. Patients were provided education and guidance from a Naturopathic doctor using a basic self-titration protocol for medical cannabis initiation. Advice on opioid tapering was provided by the pain doctor, tailored to each patient. The patients were followed in the pain department by their doctor. We report their medications, opioid milligram equivalents (OME) and pain scores, pre and post cannabis initiation.

**Results:** All six patients were successful in tapering their opioid medications.

**Discussion:** Low dose cannabis, by inhalation and/or oral dosing was tolerable and effective for facilitating opioid tapering and reduction in pain scores.

**eAppendix 2: The following general guidelines were used for issuance of the MCA to the patient:**

1. Failure of conservative treatments including PT/home exercise program/osteopathic manipulation/acupuncture
2. Trial of at least one nonopioid medication; +/- injection therapy;
3. +/- referral to pain psychologist;
4. Patient on chronic opioids with past failed attempts to wean and interest in weaning;
5. Patient on chronic opioids with noncompliance or misuse and forced into a taper;
6. Offering of MCA issued as an alternative to prescribing opioids.

**eAppendix 3: Data Dictionary for Chart Extraction**

Order for Patient Information Extract Service requested the following data for this analysis:

Demographics

Primary Diagnosis / ICD10 code (see Supplement 2)

MCA, no/yes

Dosing consultation no/yes (confirmed by records from outside clinic)

Opioid medications prescribed in UCSD healthcare system (found in EHR)

Quarters included:

July 1-Sept 30 2016

Oct 1-Dec 31 2016

Jan 1-Mar 31 2017

April 1 -June 30 2017

July1- Sept 30 2017

Oct 1- Dec 31 2017

Jan 1- Mar 31 2018

Apr 1- June 30 2018

July 1- Sept 30 2018

Oct 1- Dec 31 2018

Jan 1- Mar 31 2019

Apr 1-June 30 2019

July 1- Sept 30 2019

**eAppendix 4: General Guidelines for Dosing Consultation**

1. Patient history is reviewed in the UCSD EHR prior to time of visit.
2. Patient history of past or current cannabis use is reviewed.
3. Patient prescriptions are reviewed to assess for potential drug-drug interactions.
4. Patient use of opioids is reviewed and discussed with the patient
5. Evaluation of the patient is based on the broad systemic effects of THC on appetite, GI function, cardiovascular function, sleep disturbance, anxiety/depression, systemic inflammation and cognitive function
6. Patients may be instructed to use oral and/or dry vaporization of cannabis flower.
7. Education on types of cannabis oral products is provided.
8. Patients are instructed in a slow dose up-titration or oral cannabis over a period of 10-14 days. Starting THC dose ranged from 0.5 to 2.5 mg. Patients were advised not to administer doses over 10 mg oral cannabis.
9. Education on types of dry vape devices is provided.
10. Instructions for dry vaping of cannabis flower is provided
11. Patients are educated on the evidence for administration of low potency cannabis (5-10% THC content) for chronic pain.
12. Patients are instructed to purchase products only through cannabis dispensaries licensed by the State of California.
13. Patients are instructed on the potential side effects and/or adverse events related to cannabis intoxication.
14. Patients are provided cautionary advice related to dizziness, balance issues, change in blood pressure, driving and cognitive effects.
15. These instructions may be personalized based on patient preference, perceived tolerance to THC (based on historical use) and/or presence of acute or severe neuropathic pain.
