# Supplementary material for: Medical cannabis authorization and opioid milligram equivalents over time in patients with chronic pain: a retrospective analysis
Source: Pain Med. 2025 Aug 21;27(2):127–35. doi: 10.1093/pm/pnaf113 (PMC12865101; doi:10.1093/pm/pnaf113)
Supplement: pnaf113_Supplementary_Data [file pnaf113_supplementary_data.zip › Supplement 2.docx]

Medical Cannabis Authorization and Opioid Milligram Equivalents Over Time In Patients with Chronic Pain: A Retrospective Analysis

Michelle Sexton ND^1^, Nicholos Glodosky^2^ PhD, Carrie Cuttler PhD^2^, Michael Cleveland PhD ^2^, Euyhyun Lee^3^, Greg Polston MD^4^, Tim Furnish MD^4^, Emanuel Lerman MD^4^, Mark Wallace MD^4^, Nathaniel Schuster MD^4^

eAppendix 1 Qualifying Diagnoses: ICD10 codes for pain and neuropathic pain

eAppendix 2 Medications included in and the Calculation of OME

This supplemental material has been provided by the authors to give readers additional information about their work.

**eAppendix 1 ICD10 diagnostic codes used for chronic pain cohort**

Radiculopathy M54,

Chronic low back pain M54.5

Neck pain M54.2

Leg pain M79.906

Neck pain M54.2

Lumbar radiculopathy M54.16

Degeneration of lumbosacral intervertebral disc M51.37

Ankle pain M25.579

Lumbar spondylosis M47.816

Spinal cord injury S34.1

Spinal Stenosis M48.062

Peripheral neuropathy G90.09

Diabetic neuropathy E13.4

Major joint arthritis

Osteoarthritis of the spine M47.27

Post-laminectomy pain syndrome M96.1;

Disc disorders M51

Neuropathy G62.1

Trigeminal neuralgia G50.0 and B02.22

Facial Neuropathy G51.0

Neuralgia and neuritis, unspecified M79.2

Idiopathic peripheral autonomic neuropathy G90.09

Peripheral polyneuropathy G62.9

Other polyneuropathy G62.98

Polymyalgia Rheumatica M53.5

Fibromyalgia M79.7

Gastroparesis K31.84

Arthritis M13.8

Chronic Abdominal pain R10.9

Other Chronic Pain G89.29

Ehlers-Danlos Syndrome Q79.6

Pins and needles sensation R20.2

Lumbar spondylosis M47.816

Irritable Bowel Syndrome K59.5

Systemic Lupus Erythematosus M32.9

Thoracic Outlet syndrome G54.0

Reflex Sympathetic Dystrophy M89

Complex Regional Pain Syndrome G90.5

Post-polio syndrome G14

Rectal pain K62.89

Other chronic post-procedural pain G89.28

Abdominal Pain R10.9

Tarsal Tunnel Syndrome G57.5

Long term current use of opiate analgesic Z79.891

**Excluded:**

Paraneoplastic neuropathy G13.0

Cancer related pain G89.3

**Neuropathic Pain Diagnoses: these were agreed upon by the pain specialists from the full list shown above.**

Radiculopathy M54.1

Chronic low back pain M54.

Post laminectomy syndrome M96.1

Axial back pain M54.5

Lumbar radiculopathy M54.16

Dorsalgia M54.9

Spinal cord injury S34.1

Spinal Stenosis M48.062

Peripheral neuropathy G90.09

Diabetic neuropathy E13.4

Neuropathy G62.1

Trigeminal neuralgia G50.0 and B02.22

Facial Neuropathy G51.0

Neuralgia and neuritis, unspecified M79.2

Idiopathic peripheral autonomic neuropathy G90.0

Peripheral polyneuropathy G62.9;

Other polyneuropathy G62.98

Thoracic Outlet syndrome G54.0

Reflex Sympathetic Dystrophy M89

Complex Regional Pain Syndrome G90.5

Post-polio syndrome G14

Tarsal Tunnel Syndrome G57.5

**eAppendix 2: Calculation of OME**

Medication orders included the dose, unit, sign order status included

prescriptions for the following opioid were calculated:

Codeine

Fentanyl

Hydrocodone

Hydromorphone

Methadone

Oxycodone

Morphine

Oxymorphone

Prescriptions labeled in the electronic health record as "NULL", "COMPLETED", "SUSPEND", "DISPENSED" or "VERIFIED", with a missing end date or dose, or a prescription for a single day duration (as for a procedure), were excluded from the analysis.

(These prescriptions were mistakes, cancellations or associated with a procedural or emergency department visit.)

The equation for conversion of prescribed opioid to OME over time:

(total quantity of pills ordered) x (milligrams of drug converted to OME) = total milligrams prescribed (per quarter)

(duration of the prescription (# of days : end date – start date)

= OME/day/quarter

Calculations that resulted in an OME value that was negative were calculated when the ‘end date’ prescription was before the ‘start date’ (system errors).

*These calculations with negative values were excluded from analysis.

All missing values for the OME variable were assumed to be zero.

This was interpreted as the patient never received an opioid prescription for a specific quarter.
